# 

**Published:** 1878-07

**Authors:** 


### Books and Pamphlets Received.

Physics of the Infectious Diseases. By C. A. Logan, A. M., M. D. Chicago: Jansen, McClung & Co., 1878.

Atlas of the Diseases of the Skin. By Balmanno Squire, M. B. London: J. & A. Churchill, 1878.

Intra-Venous Injections of Milk. By T. Gaillard Thomas, M. D. (Reprint from New York Medical Journal. New York: D. Appleton & Co., 1878.

Laparo-Elyilotomy. By T. Gaillard Thomas, M. D. New York: Wm. Wood & Co., 1878.

The London Lancet, June, 1878.

Annual Report of Eye and Ear Department of St. Mary's Hospital, Detroit.

Quarterly Journal of Inebriety,

Popular Science Monthly, July, and Supplement No. XIV.

Announcements of Bellevue Hospital Medical College; University of Louisiana Medical Department; and Albany Medical College.

An open letter to Eugene Grissom, M. D. LL. D. By Wm. A. Hammond, M. D.

Fluid Extracts by Repercolation. By E. R. Squbb, M. D.

A Contribution to the Pathological Anatomy of Desseminated Cerebro-Spinal Sclerosis. By Dr. E. C. Seguin, New York; Dr. J. C. Shaw, Brooklyn, and Dr. A. Van Derveer, Albany.

Post-Paralytic Chorea. By E. C. Seguin, M. D.

Localized Cerebral Lesions. By E. C. Seguin, M. D.

### INDEX.

**A**

Acne, Treatment of, ... 430
Address of Prof. F. H. Hamilton at Woman's Hospital, ... 306
Adenitis, Acute and Chronic. By J. R. Larzelere, M. D., ... 204
Alcohol, Influence of, ... 109
American Medical Association, Proceedings of Twenty-Eighth Annual Meeting. 25
Proceedings of the Twenty-Ninth Annual Meeting, ... 451
American Medical College Association, ... 467
American Medical Editors Meeting of, ... 470
Amyl Nitrite, ... 350
Andrews, J. B., A. M., M. D. Purpura occurring in Nervous Disease, ... 401
Angular Curvature of the Spine. Clinical Lecture. By Prof. J. F. Miner, M. D., ... 49
Antiseptic Surgery, ... 137
Aphorisms in Fracture. By Richard O. Cowling. A. M., M. D., ... 297
Are Ants Civilized? ... 222
Arsenic in Wall Papers and Dresses, ... 221
Arsenic Poisoning of the River Rhine averted, ... 903
Ayer, George, M. D., Erie County Medical Society Meeting and Memorial on the Death of, ... 231

**B**

Balsam Peru, Therapeutic Action of, ... 260
Barnes, Edwin R., M. D. Morbus Coxarius, 321
Baths, Continuous in Vienna General Hospital, ... 384
Berlin, Letter from. By Chas F. A. Nichell, M. D., ... 270
Belladonna Tincture, Externally in Night-Sweats, ... 385
Books and Pamphlets Received, ... 40, 80
120, 160, 198, 240, 280, 320, 359, 400, 440, 480

Books Reviewed.

A Compend of Diagnosis in Pathological Anatomy. By Johannes Orth, Translated by F. C. Shattuck, M. D., and G. K. Sabine, M. D. and revised by R. H. Fitz, M. D., ... 279
Address before the Rocky Mountain Medical Association. By J. M. Toner, M. D., ... 399
Advantages and Accidents of Artificial Anesthesia. By Lawrence Turnbull, M. D., ... 438
A Guide to Therapeutics and Materia Medica. By Robert Farquharson, M. D., Edin., F. R. C. P., ... 358
A Practical Treatise on Materia Medica and Therapeutics. By Roberts Bartholow, A. M., M. D., ... 39

Books Reviewed Continued.

Atlas of Skin Diseases. By Louis A. Duhring, M. D., ... 117
A Treatise on Gonorrhœa and Syphilis, 359
Biology, with Preludes on Current Events. By Joseph Cook, ... 198
Civil Malpractice. A Treatise upon Surgical Jurisprudence. By Milo A. McClelland, M. D., ... 75
Cutaneous and Venereal Memoranda. By Henry G. Piffard, A. M., M. D., and George Henry Fox. A. M., M. D., 238
Diseases of the Skin, A Practical Treatise on. By Louis A Duhring, M. D., 159
Diseases of the Skin, Elementary Treatise on. By Henry G. Piffard, A. M., M. D., ... 118
Elements of Therapeutics. By C. Binz, M. D., ... 439
Excisions of the larger Joints of the Extremities. Prize Essay Supplement. Vol. XXVII. of the Transactions of the American Med. Association. By H. Culbertson, M. D., ... 153
Eye. Its injuries and their Medico-Legal Aspect, ... 479
Faradaic and Galvanic Currents in the Electro-thermal Bath, with History of Cases By Justin Hayes, M. D., ... 119
Forensic Medicine and Toxicology. By W. B. Woodman, M. D, F. R. C. P. and Chas. M. Tidy. M. B., F. C. S., ... 194
Hand-Book of the Practice of Medicine. By M. Charteris, M D., ... 889
Hand-Book for Hospital Visitors, ... 40
Hospitals; Their History, Organization and Construction, Boylston Prize Essay. By W. Gill Wylie, M. D., ... 158
How to use the Ophthalmoscope. By Edgar Brown, Surgeon to the Liverpool Eye and Ear Infirmary, ... 40
Index of Diseases and their Treatment. By Thos. H Tanner. M. D., F. L. S., 196
Landmarks Medical and Surgical. By Luther Holden. F. R. C. S., ... 438
Lectures on Fevers. By Alfred L. Loomis, A. M., M. D., ... 237
Lectures upon Practical Surgery. By H. H. Toland, M. D., ... 194
Materia Medica for the use of Students. By John B. Biddle, M. D., ... 356
Medical Reform, General Ideas concerning. By David Hunt, M. D., ... 119
Microscope, Practical Hints on the use of. By John Phen, ... 158
Modern Medical Therapeutics. By Geo. H. Napheys, A. M., M. D., ... 319
Modern Surgical Therapeutics. By Geo. H. Napheys, A. M., M. D., ... 319

BOOKS REVIEWED CONTINUED.

Outlines of Modern Chemistry. By C. Gilbert Wheeler, ...... 239
Personal Appearance and the Culture of Beauty. By T. S. Sozinsky, M. D., Ph. D.,...... 239
Practical Gynecology. By Haywood Smith, M. A., M. D. Oxon.,...... 437
Prescription Writing, by F. H. Gerrish, M. D., ...... 478
Practitioners Reference Book. By Richard J. Dunglison, M. D.,...... 196
Public Hygiene in America. By Henry I. Bowditch, M. D.,...... 319
Surgical Observations, with cases and Operations. By J. Mason Warren, M. D.,...... 160
The action of Medicine. By Isaac Ott, A. M., M. D., ...... 357
The Cure of Rupture, reducible and irreducible, also of Vericocele and Hydrocele, by new Methods. By George Heaton, M. D.,...... 34
The Ear; its Anatomy, Physiology and Diseases. By Chas. H. Burnett, A. M., M. D.,...... 195
The Pathology and Treatment of Childbed. By Dr. F. Winckel. Translated from the German by James R. Chadwick, M. D., ...... 37
The Question of Rest for Women during Menstruation. Boylston Prize Essay. By Mary Putnam Jacobi, M. D.,...... 32
The Science and Art of Surgery. By John Eric Erichsen, F. R. S.,...... 399
Transactions of the American Gynecological Society,...... 115
Transactions of the American Medical Association, Volume XXVIII., ...... 398
Transactions of the College of Physicians of Philadelphia. Vol. X.,...... 157
Transactions of the International Medical Congress at Philadelphia, 1877,...... 276
Transactions of the Kentucky State Medical Society,...... 157
Transactions of the Medical Society of the State of New York for 1877,...... 439
Transactions of the Medical Society of the State of Pennsylvania at its Twenty-Eight Annual Session,...... 357
Transactions of The New York Pathological Society. Volume I.,...... 39
Volume II.,...... 238
Vest Pocket Anatomist. By C. Henri Leonard, M. D.,...... 440
Wood's Physician's Vade Mecum and Visiting List. Arranged by H. C. Wood, M. D.,...... 196
Ziemssen's Cyclopædia of the Practice of Medicine. Vol. XII. Diseases of the Brain and its Membranes,...... 77
Vol. VIII. Diseases of the Chylopœtic System, ...... 114
Vol. XV. Diseases of the Kidney,...... 154
Vol. XVI. Diseases of the Locomotive Apparatus. Anomalies of Nutrition. 236
Vol. XIV. Disease of the Nervous System,...... 476

Brush, Edward N., M. D. Report of case of Amputation of Cervix Uteri with Galvano-Cautery Apparatus, by Prof. James P. White, M. D.,...... 201
Buffalo Medical Association, Abstract of Proceedings of,...... 10, 96, 133, 332, 376, 419
Burns, Bicarbonate of Soda Dressing for,...... 435

C

Catgut Sutures in Surgical Operations,...... 472
Cervix Uteri, Epithelioma of, with Amputation by Galvano-Cautery. By Prof. Jas. P. White, M. D.,...... 101
Chancres, Hard, Excision of,...... 429
Chapman, E. N., A. M., M. D. Treatment of Diphtheria,...... 81
Charities, Medical, Abuse of,...... 177
Chlorate ot Potash, Poisoning by, ...... 431
Cicatrization of Large Wounds under the so-called Bordeaux dressing,...... 145
Clarke, Prof. Edward H., M. D. Obituary of,...... 230
Code of Ethics,...... 267
Composition of Secret Remedies,...... 227
Compressed Air, New Treatment for the Administration of,...... 185
Constipation, Cascara Sagrado in,...... 349
CORRESPONDENCE
By S. P. Radcliffe, M. D.,...... 255
From A. D. A. King, M. D.,...... 296
From Chas. F. A. Nichell, M. D.,...... 290
From H. B. Osborne, M. D.,...... 293
From Nil Desperandum,......59, 98, 175, 336
From Prof. Frank H. Hamilton, M. D., 256
From Prof. James P. White, M. D.,..257, 340
From S. G. Dorr, M. D.,...... 423
Club Foot, Clinical Lecture on. By Prof. J. F. Miner, M. D.,...... 281
Cronyn's Boudoir Medicines,...... 274, 296
Croup, Temperature in,...... 346
Curtis, F. C., M. D. Certain Points in Diphtheria, ...... 41

D

Dean, Henry W., M. D. Death of, and Obituary,...... 275
Delirium Tremens, Capsicum in,...... 386
Dialyzed Iron,...... 182
in arsenical poisoning,...... 313, 387
Diphtheria, Certain Points in. By F. C. Curtis, M. D.,...... 41
Croup and Tracheotomy, ...... 186
Etiology of,...... 262
In report of California State Board of Health,...... 270
Treatment of. By E. N. Chapman, A. M., M. D.,...... 81

E

Ear, Foreign Bodies in,...... 428
EDITORIAL.
Advertising by Physicians,...... 73
American Medical Association, General Notice of,...... 443, 474
Announcement of Secretary,...... 391
Announcement of Surgical Section of,. 351
Buffalo Medical College Commencement and Meeting of Alumni,...... 315
Charities of Physicians,...... 150
Education,...... 111
Lactopeptine, ...... 193
Medical Legislation,...... 31
Modern Homœopathy,...... 192
New Dispensaries,...... 272
Opening of Term at Buffalo Medical College,...... 193
Struggle for Existence,...... 232
Transactions of the Alumni Association, 73
Elementary Advice to Mothers and Nurses, 218
Epilepsy, Cure of,...... 350
Epilepsy, Treatment of...... 389

Erie County Medical Society. Meeting of and Memorial on the Death of Prof. Geo. Hadley, M. D., 446
Memorial on Death of Geo. Ayer, M. D., 231
Eucalyptus, 383
Eve, Prof. Paul F. M. D., Obituary Notice, 187
Exchanges, List of, 199
External Glandular Enlargement. The Physiology of the Lymph System of Human Bodies. By Z. C. McElroy, M. D., 168

**F**

Femur, Shaft of, Summary of remarks by Prof. F. H. Hamilton, on Fractures of, in Adults. By James C. Lay, M. D., M. R. C. S. E., 173
Ferrum Dialysatum, 182
Fetal Auscultation, Death of Discoverer of, 388
Forceps, The Obstetrical. By Prof. Jas. P. White, M. D. Reported by Joseph Fowler, M. D., 408
Fracture, Aphorisms in, 297

**G**

Gastrotomy, 345
Gay, Chas. C. F., M. D. Model Pocket Case of Instruments, 334
Germ Theory Controversy, 67
Glycosuric Re-agent, New, 431

**H**

Hadley, Prof. George, M. D., Memorial Session of Erie County Society, and Medical Students Memorial upon the Death of, 146
Tribute to Memory of, by Prof. Thos F. Rochester, M. D, in Lecture Introductory to the Buffalo Medical College Course, 188
Hamilton, Prof. Frank H., Letter from, 256
Hamilton, Prof. F. H. Address at Woman's Hospital, 306
Hay Fever, 110
Head, Inquiries of, 427
Hemorrhage, Post Partum, treated by Injection of Hot Water, 428
Hæmoptysis, Treatment of, 258
Hubbell, A. M., M. D., A case of Chronic Intussusception of Eleven Months standing, 328
A Case of Intussusception, in which Laparotomy was performed, 241
Huxley, Prof., On the Anti-Vivisectionists, 109
Humerus, Fracture of, with dislocation, Head removed by Dr. J. Cronyn. Reported by F. Peterson, 449

**I**

Ileum. Separation of and Spontaneous Occlusion of the divided Extremities. By Prof. Thomas F. Rochester, M. D., 94
Insane, Cost of Maintenance of, 62
Infant Mortality and Infant Food 106
International Congress of Medical Sciences at Geneva, 1877, 223
Intestinal Obstructions, Symptoms in 348
Intussusception, A Case, in which Laparotomy was performed. By A. A. Hubbell, M. D., 241
A Case of, Chronic, of Eleven Months standing. By A. A. Hubbell, M. D., 328
Iron, Contra-indicated, 385

**J**

Japanese, Children's Disorders among, 264
Jaw, Fracture of Lower, in an Infant. By Geo. A. Mursick, M. D., 295

**K**

Kidney, Extirpation of, 263
Knee Joint, Indications for Drainage of, 432

**L**

Laparotomy, A Case of Intussusception. By A. A. Hnbbell, M. D., 241
Larzelere, J. R., M. D. Adenitis, Acute and Chronic, 204
Larynx, Excision of, 350
Lay, James C., M. D., M. R. C. S. E. Summary of Remarks by Prof. Frank H. Hamilton, M. D., upon Fractures of the Shaft of the Femur, 173
Lester, Ellwood C., M. D Extract of Malt and Phosphorus Compounds in the Treatment of Phthisis 383
Library, United States Medical, 230
Limitations of the Senses, 432

**M**

Malaria, Prevalence of, 424
Malt, Extract and Phosphorus Compounds in the Treatment of Phthisis. By Ellwood C. Lester, M. D., 373
Materia Medica and New Remedies. By Thomas D. Washburn, M. D., 161
McElroy, Z. C., M. D. External Glandular Enlargements. Physiology of the Lymphatic System, 168
Median Lithotomy. By Chas A Wall, B. S., 361
Medical Certificates in the Daily Papers, 215, 266
Medical Experts as Witnesses in Courts of Justice, 378
Medical Notes and Comments, 352, 394, 434
Medical Reform, 108
Medico-Legal Facts and Testimony in the Trial of Henry C Hendryx for the murder of his wife. By J. C. Young, M. D., 57
Midwifery, Early Rupture of the Membranes in, 259
Military Surgery in Turkey, 230
Milk, Intra-venous Injection of, 426
Miner. Prof. J. F., M. D. Clinical Lecture upon Angular Curvature, 49
do upon Club Foot, 281
do upon Spinal Curve, 1
do upon the Plaster of Paris Jacket in Spinal Disease, 254
Operation for Recto-Vaginal Fistula, 370
Removal of the Thyroid Gland, 207
Morbus Coxarius. By Edwin R. Barnes, M. D., 321
Mursick, Geo. A., M. D., Fracture of Lower Jaw in an Infant treated with Wire Sutures, 295

**N**

Nature of Communicable Diseases, 184
Near-Sighted, Is the Eye becoming Myopic under the influence of Modern Education, 261
Newspaper Law Decisions 153
Nichell, Chas. F. A., M. D. Letter from Berlin, 290
Treatment of Placenta Prævia, 415
Nursing in Columbia, 265

**O**

Obstetrical Society of London, 18
Obstetrics and Gynæcology, Recent Progress in, 69
Opium, Antidotes, 110
Osborne, H. B., M. D. Letter from, 293
Ovariotomy at Samaritan Hospital, 388
Ovariotomy during Pregnancy, 187
Oxygen, Liquefaction of, 278

**P**

Peaslee, Prof. E. R., M. D. Obituary of,.. 317
Peterson F. Report of a case of Epiphysial Fracture of Humerus,.................. 449
Pimento, Extract of, as a revulsion,........ 350
Placenta Prævia, Its Treatment. By Chas. F. A. Nichell, M. D ,..................... 415
Plaster of Paris Jacket in Spinal Diseases, 210
Plaster of Paris Jacket in Spinal Disease, Clinical Lecture upon. By Prof. J. F. Miner, M. D.,.......................... 254
Pocket Case of Instruments, Model. By Chas. C. F. Gay, M. D.,.................. 334
Poison of Putrefied Blood, ................ 219
Popular Science Monthly,.............. 236, 436
Potter, Prof. Milton G., M. D. Obituary,.. 316
Professional Overwork,.................... 347
Prophylactic Treatment of Placenta Prævia 220
Purpura occurring in Nervous Disease, By J. B. Andrews, M. D.,............... 401
Pyo-Pneumo Thorax. Treatment by Aspiration and permanent Drainage tube. By Prof. E. V. Stoddard, M. D.,............. 131

**Q**

Quarantine, Intelligent and Efficient, ...... 270
Quinia, Cinchonia and Cinchonidia, Comparative Action of,.......................... 107

**R**

Radcliffe, S. J., M. D. Correspondence with Prof. Frank H. Hamilton, M. D.,........ 255
Recto-Vaginal Fistula. Operation by Prof. J. F. Miner, M. D......................... 370
Rheumatic Fever, treated by Salicylic Acid, 341
Rochester, Prof. Thomas F., M. D., Separation of the Ileum and Spontaneous Occlusion of the Divided Extremities,...... 106
Tribute to the Memory of Prof. George Hadley, M. D., in Lecture Introductory to Buffalo Medical College Course,....... 188
Rush Medical College and Homœopothy,.. 274

**S**

Salicylic Acid in Rheumatism,........... .. 214
Sclerotic Acid, ......... ................. 264
Sleeplessness, Treatment of,............... 348
Spinal Curve, Clinical Lecture upon By Prof. J. F. Miner. M. D.,................ 1
Stockton Charles G., M. D. Report of Operation for Recto-Vaginal Fistula by Prof. J. F. Miner. M. D.,................ 370
Stoddard, Prof. E. V., M. D. Pyo-Pneumo-Thorax. Treatment by Aspiration and permanent Drainage Tube., .......... ... 131
Surgical Reports. By Chas. A. Wall, B. S., 122
Suspension in Spinal Disease,.... ........ 347
Syphilis, Mercurial Treatment of,.......... 384

**T**

Temptations of Physicians,................ 429
Tetanus, Treatment of,.................... 386
Therapeutist, A daring,......... .......... 349
Thyroid Gland, Removal of. By. Prof. J. F. Miner. M. D.,......................... 207
Typhoid Fever, Poison of conveyed by Water,................................ 258
Typhoid Infection,........................ 183
Tucker, Prof. W. G., Urea, Quantative Analysis of,..... ............................ 441

**U**

Ureter, Accidental Section of during Ovariotomy,... .............................. 30
Urethra, Dilatation of the Urine itself...265 293
Uterus Inverted for Forty Years, Reduced in half an hour by a new method......... 381
Uterus, Inverted, spontaneous reduction of, 105
Uterus, Retroflexion of, New mode of treating,.................................... 19

**V**

Vaginal Speculum, New. By S. G. Dorr, M. D.,..... ................ ............ 423
Variola and Varicela, Case in Proof of Non-Identity of,............................ 228
Vinegar, Dangerous, ....................... 108

**W**

Wall, Charles A., B. S., Medical Notes and Comments.................... 352, 394, 434
Correspondence,......................... 338
Median Lithotomy,...................... 361
Report of Case of Removal of Thyroid Gland, By Prof. J. F. Miner, M. D.,...... 207
Surgical Reports,....................... 127
Washburn, Thomas A., M. D., Materia Medica and New Remedies,.............. 169
White, Prof. James P., Amputation of Cervix Uteri with Galvano-Cautery for Epithelioma,......................... .. 201
Letter from, ............................ 257
The Obstetrical Forceps,................ 409
Whooping Cough, Carbolate of Soda in,... 381
Witnesses, Medical Experts as, ........ ... 378
Medical, Proper Compensation to,.. 229, 340
Women in the Profession,................. 102

**Y**

Young, J. C., M. D. Medico-legal Facts and Testimony in the Trial of Henry C. Hendryx for the murder of his wife,.... 57

